# ANTP-SmacN7融合肽对H460细胞的辐射增敏作用

**DOI:** 10.3779/j.issn.1009-3419.2016.05.01

**Published:** 2016-05-20

**Authors:** 宝娜 刘, 利清 杜, 畅 徐, 彦 王, 芹 王, 志仪 宋, 晓辉 孙, 津晗 王, 强 刘

**Affiliations:** 1 300192 天津，中国医学科学院北京协和医学院放射医学研究所，天津市放射医学与分子核医学重点实验室 Institute of Radiation Medicine of Chinese Academy of Medical Science and Peking Union Medical College, Tianjin Key Lab of Radiation and Molecular Nuclear Medicine, Tianjin 300192, China; 2 300120 天津，天津市中医药研究院附属医院 Tianjin Academy of Traditional Chinese Medicine Affiliated Hospital, Tianjin 300120, China

**Keywords:** Smac蛋白, 肿瘤细胞, 细胞凋亡, 辐射敏感性, Smac protein, Tumor cell, Apoptosis, Radiosensitivity

## Abstract

**背景与目的:**

肿瘤的辐射耐受制约了放疗疗效，第二个线粒体衍生的半胱氨酸蛋白酶激活剂（Second mitochondria-derived activator of caspase, Smac）蛋白类似物可明显提高辐射诱导的肿瘤细胞凋亡，有望成为新型肿瘤辐射增敏药物。本研究旨在探讨新型Smac蛋白类似物ANTP-SmacN7融合肽对肺癌细胞系H460的辐射增敏作用。

**方法:**

合成ANTP-SmacN7融合肽，连接荧光素FITC以观察融合肽能否进入细胞。对数生长期H460细胞分为空白对照组、单纯照射组、ANTP-SmacN7组和照射联合ANTP-SmacN7组，单纯照射组给予0 Gy、2 Gy、4 Gy、6 Gy照射，照射联合ANTP-SmacN7组中ANTP-SmacN7的浓度为20 μmol/L，WST-1测定H460细胞的增殖。流式细胞仪测定细胞处理后24 h和48 h的细胞凋亡率。Western blot实验检测caspase3和cleaved caspase3的表达水平。

**结果:**

ANTP-SmacN7融合能够顺利进入细胞，且能够增强H460细胞的辐射敏感性（*F*=25.1，*P* < 0.01，增敏比为1.86），照射联合ANTP-SmacN7可明显降低H460细胞的克隆形成率（*χ*^2^=45.2, *P* < 0.01; *χ*^2^=40.3, *P* < 0.01），提高cleaved caspase3的表达量，促进caspase3的活化，增加辐射诱导的细胞凋亡率。

**结论:**

ANTP-SmacN7融合肽可明显提高H460细胞的辐射敏感性，作为一种新的Smac蛋白类似物有望用于肿瘤的辐射增敏治疗。

## Abstract (en)

**Background and objective:**

The curative effect of radiotherapy may be limited by the radioresistance of tumor. Mimetic compounds of Second mitochondria-derived activator of caspase (Smac) were hopeful to become new drugs of radiosensitization for tumor because they can increase radiation induced apoptosis in tumor cells. The aim of present study is to observe the radiosensitization effect of a new Smac mimetic ANTP-SmacN7 fusion peptide in H460 cell line.

**Methods:**

In order to observe if the fusion peptide can enter into tumor cell, ANTP-SmacN7 fusion peptide was synthesized and linked by FITC. H460 cell was divided into control, radiation only, ANTP-SmacN7 only and ANTP-SmacN7 combined with radiation group. The cells were exposed by 0, 2, 4 and 6 Gy and the concentration of ANTP-SmacN7 was 20 μmol/L. Proliferation of H460 tumor cell was detected by WST-1 assay. There are four groups in the present study: control group, radiation group, ANTP-SmacN7 group and ANTP-SmacN7 combined with radiation group. Apoptosis was detected by flow cytometry at 24 and 48 hours after the treatment of all the groups. The level of caspase3 and cleaved caspase3 were detected by Western blot assay.

**Results:**

ANTP-SmacN7 can enter into cells and promote the radiosensitization of H460 cell obviously (*F*=25.1, *P* < 0.01, sensitivity enhancement ratio was 1.86). The treatment of ANTP-SmacN7 combined with radiation decreased the cloning forming efficiency (*χ*^2^=45.2, *P* < 0.01; *χ*^2^=40.3, *P* < 0.01), activated caspase3 by promoting the expression of cleaved caspase3 and increased the apoptosis of H460 cell line.

**Conclusion:**

ANTP-SmacN7 fusion peptide had remarkably radiosensitization effect on H460 cell line. ANTP-SmacN7 fusion peptide might be hopeful to be applied in radiosensitization therapy as a new Smac mimetic polypeptide in the future.

放射治疗在肿瘤治疗中的作用和地位日益突出，已成为治疗恶性肿瘤的主要手段之一。然而，肿瘤细胞在放疗过程中的辐射耐受成为限制放疗疗效的主要问题。因此，肿瘤细胞的辐射增敏研究成为近年来的研究热点。第二个线粒体衍生的半胱氨酸蛋白酶激活剂（Second mitochondria-derived activator of caspase, Smac）作为一种促凋亡蛋白^[1]^，当受到凋亡诱导因子（抗癌药物、电离辐射、紫外线、化学信号和DNA损伤等）的作用时，可以和其他线粒体蛋白如细胞色素-c等穿过线粒体膜释放至胞浆中，参与凋亡调节^[2, 3]^。Smac是目前发现的唯一一种在哺乳动物体内能直接抑制凋亡抑制蛋白（inhibitors of apoptosis proteins, IAPs）的分子^[4]^，Smac氨基端的7个氨基酸残基SmacN7是Smac蛋白中最小的活性单位，能够抑制IAPs发挥辐射增敏作用，但SmacN7无法顺利进入细胞^[5]^。研究^[6]^表明，果蝇Antennapedia蛋白（ANTP）同源结构域的第三个α螺旋（43-58之间的16个氨基酸残基）是具有转导功能的最小蛋白区域，不依赖于受体、通道、能量及胞吞作用，可以直接作用于脂质双分子层完成跨膜运动而进入细胞。因此，我们选择ANTP蛋白作为引导肽，合成ANTP-SmacN7融合肽，借助引导肽的作用，把SmacN7引导进入细胞，使其发挥辐射增敏作用。

## 1 材料与方法

### 1.1 材料和主要试剂

肺癌细胞H460细胞株为放射医学研究所辐射损伤效应室实验室保存。胰蛋白酶、乙二胺四乙酸（EDTA）裂解液购自美国Gibco公司；Annexin V凋亡试剂盒购自美国BD生物科学公司；10%胎牛血清（FBS）购自美国Gibco公司；RPMI 1640培养基购自美国Gibco公司；磷酸盐缓冲液（phosphate buffered saline, PBS）为放射医学研究所辐射损伤效应室实验室配制；正置荧光显微镜购自日本尼康公司；流式细胞仪购自美国贝克曼公司。

### 1.2 多肽合成以及多肽的细胞渗透试验

以ANTP蛋白的ANTP9肽作为引导肽，Smac7作为功能肽，合成ANTP-SmacN7融合肽（上海生工生物工程有限公司完成）。为了观察该融合蛋白是否具有细胞渗透的功能，在其羧基端用异硫氰酸荧光素（FITC）进行标记。细胞培养过程中加入ANTP-SmacN7，浓度为20 µmol/L。继续培养3 h后，荧光显微镜观察融合肽进入细胞情况。

### 1.3 WST-1生长实验

将对数生长期的细胞，用胰酶消化后，用含10%胎牛血清的培养基配成浓度为2.5×10^4^个/mL的细胞悬液。接种在96孔培养板中，每孔200 µL细胞悬液，设5个平行孔。经37 ℃、5%CO_2_条件下培养12 h待细胞贴壁。不同条件处理细胞后，到指定时间取出细胞，将孔内细胞培养基轻轻吸出，每孔加WST-1 20 µL，放入孵箱继续培养0.5 h-4 h。在多功能酶标仪上用450 nm波长测定吸光度值（*A*）。根据吸光度值（*A*）计算细胞存活率。细胞存活率=（实验孔*A*值-空白孔*A*值）/（对照孔*A*值-空白孔*A*值）×100%。

### 1.4 Annexin V-PITC/PI双染法测定细胞凋亡

细胞处理后24 h和48 h检测细胞凋亡。细胞用不含EDTA的胰酶消化收集；用PBS洗涤细胞2次，1, 500 r/min离心5 min，离心半径5 cm，收集5×10^5^个细胞；加入500 μL的结合缓冲液悬浮细胞；加入5 µL Annexin V-FITC混匀后，加入5 μL碘化丙啶（PI），混匀；室温、避光、反应5 min-15 min；在1 h内，进行流式细胞仪的观察和检测。激发波长Ex=488 nm；发射波长Em=530 nm。Annexin V-FITC的绿色荧光通过FITC通道（FL1）检测；PI红色荧光通过PI通道（FL2或FL3）检测。

### 1.5 克隆形成实验

以每孔400个细胞接种于6孔培养板，贴壁培养24 h后加入ANTP-smacN7继续培养24 h，随后于室温空气中经^137^Cs γ源（加拿大原子能公司，CAMMA-CELL40）照射4 Gy，源靶距30 cm，剂量率0.793 Gy/min，照后37 ℃继续培养，10 d后终止培养，固定细胞，染色。实验重复2次。细胞数≥50定义为1个克隆集落。克隆形成率（%）=克隆集落数/接种细胞数×100%。根据多靶单击方程拟合细胞存活曲线，求曲线参数D0值，计算放射增敏比（sensitivity enhancement ratio, SER）=单纯照射组D0值/加药照射组D0值。

### 1.6 Western blot实验

细胞生长至亚融合状态时收集细胞，以MER-Pierce裂解液裂解细胞。蛋白定量按BCA蛋白定量试剂盒说明书（日本Takara公司）进行。取50 μg蛋白进行不连续聚丙烯酰胺凝胶电泳，电转移至聚偏二氟乙烯膜，用含5%脱脂奶粉的TBST 4 ℃封闭过夜，加入一抗，室温孵育2 h，洗膜，加入相应的辣根过氧化物酶标记的二抗，室温孵育1 h，ECL化学发光法检测。以β-肌动蛋白作为内参^[7, 8]^。

### 1.7 统计学方法

实验结果采用SPSS 13.0统计软件进行分析。计量资料组间差异比较采用析因设计的方差分析，计数资料的比较采用卡方检验。CELSURrad98软件计算药物增敏比并绘制细胞的存活曲线。*P* < 0.05为差异有统计学意义。

## 2 结果

### 2.1 ANTP-SmacN7融合肽进入细胞

用DAPI溶液染色作为对照组，确定细胞核的位置，结果表明，ANTP-SmacN7融合蛋白可以顺利进入细胞，为融合肽在细胞内发挥促凋亡作用打下基础（图 1）。

**1:**
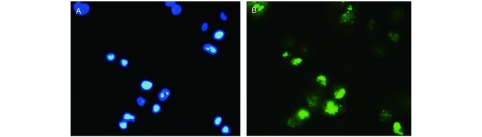
ANTP-SmacN7进入细胞。A：DAPI对照组（进行细胞核定位）；B：FITC-ANTP-SmacN7组（融合肽进入细胞）。DAPI着色（蓝色）的细胞为活细胞，FITC着色（绿色）的细胞为多肽进入细胞。 ANTP-SmacN7 enter into tumor cells. A: DAPI control group (location of cell nucleus); B: FITC-ANTP-SmacN7 group (ANTP-SmacN7 fusion peptide entered into the cells). DAPI colored (blue) cells were living cells, FITC colored (green) cells showed the peptide entered into the tumor cells

### 2.2 ANTP-SmacN7对H460细胞系的辐射增敏作用

取对数生长期H460细胞分为单纯照射组和照射联合ANTP-SmacN7组，单纯照射组给予0 Gy、2 Gy、4 Gy、6 Gy照射，照射联合ANTP-SmacN7组中ANTP-SmacN7的浓度为20 μmol/L^[9]^，ANTP-SmacN7能够增强H460细胞的辐射敏感性（*F*=25.1, *P* < 0.01），照射联合融合肽组的克隆形成率明显降低，联合组分别与ANTP-SmacN7组和单纯照射组相比，差异有统计学意义（*χ*^2^=45.2, *P* < 0.01; *χ*^2^=40.3, *P* < 0.01）。照射+ANTP-SmacN7组和照射组的D0值分别为9.61和17.87，SER为1.86。由图 2可见，ANTP-SmacN7融合肽联合照射时发挥了辐射增敏作用。

**2:**
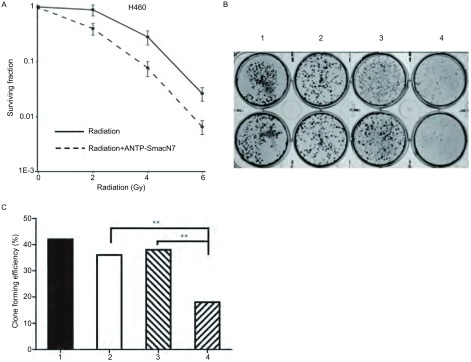
ANTP-SmacN7提高H460细胞的辐射敏感性。A：单纯照射组和联合组的存活分数，可见ANTP-SmacN7提高H460细胞的辐射敏感性，增敏比为1.86；B：克隆形成实验，1：未处理组；2：单纯照射组；3：ANTP-SmacN7组；4：照射+ANTP-SmacN7组；C：对应各组的克隆形成率。^**^P < 0.01 ANTP-SmacN7 increased the radiosensitivity of H460 cell line. A: Survival fraction of radiation and radiation+ANTP-SmacN7 group. The radiosensitivity of H460 cells were increased by ANTP-SmacN7, SER was 1.86; B: Clone forming assay, 1: Control group, 2: Radiation group, 3: ANTP-SmacN7 group, 4: Radiation+ANTP-SmacN7 group; C: Clone forming efficiency of the four groups accordingly. ^**^P < 0.01

### 2.3 ANTP-SmacN7融合肽的促凋亡作用

图 3显示H460细胞在24 h和48 h的细胞凋亡水平，联合组分别与ANTP-SmacN7组和单纯照射组相比，差异有统计学意义（24 h: *F*=42.3, *P* < 0.01; *F* =31.5, *P* < 0.01; 48 h: *F*=49.2, *P* < 0.01; *F*=35.6, *P* < 0.01）。ANTP-SmacN7单独作用时对细胞的凋亡作用不是很明显，随着时间的增加引起48 h的凋亡率比24 h的凋亡率有微小增加，照射组也出现随时间增加的凋亡。在联合组，ANTP-SmacN7的促凋亡作用在辐射的基础上明显增高。这个结果也进一步证实了ANTP-SmacN7有明显的辐射增敏作用。由图 3B可见，联合组的cleaved caspase3明显增加，表明ANTP-SmacN7联合照射促进了caspase3的活化。

**3:**
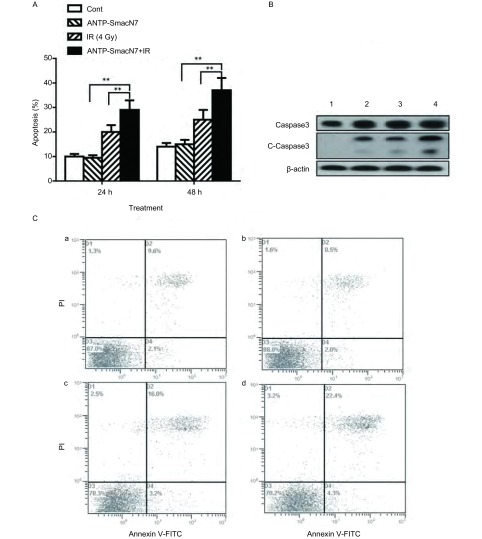
ANTP-SmacN7对H460细胞凋亡及caspase3表达量的影响。A：各组细胞在处理后24 h和48 h的细胞凋亡率；B：各组细胞caspase3和cleaved caspase3（C-Caspase3）表达量的变化。1-4依次为：对照组、ANTP-SmacN7组、单纯照射组和ANTP-SmacN7联合照射组；C：H460细胞在不同处理后24 h的细胞凋亡。a：对照组；b：ANTP-SmacN7处理组；c：照射组；d：ANTP-SmacN7联合照射组。^**^P < 0.01；*n*=3 Effect on apoptosis and caspase3 expressions in H460 cells by ANTP-SmacN7. A: Apoptosis of the four groups at 24 h and 48 h after treatment; B: The expression of caspase3 and cleaved caspase3 (C-Caspase3). 1: Control group, 2: Radiation group, 3: ANTP-SmacN7 group, 4: Radiation+ANTP-SmacN7 group; C: Apoptosis of different group at 24 h after treatment for H460 cell line. a: Control group, b: Radiation group, c: ANTP-SmacN7 group, d: Radiation+ANTP-SmacN7 group. ^**^P < 0.01;*n*=3

## 3 讨论

目前研究^[10, 11]^表明，许多肿瘤的治疗和癌症患者生存率的提高，很大程度上取决于靶向肿瘤细胞的治疗抗性和新型抗癌药物的识别，其主要机制就是促进肿瘤细胞的凋亡，包括化疗、放疗、免疫治疗和细胞因子治疗。然而肿瘤细胞在放疗过程中产生的辐射抗性往往限制了放疗的远期疗效，其机制与IAPs的高表达有关。因此，找到一种可以通过抑制IAPs并促进细胞凋亡而提高辐射敏感性的途径事关重要。在本研究中，我们首先确定了放射增敏活性小分子ANTP-SmacN7^[12]^，含有一个促进凋亡序列，可以破坏凋亡抑制蛋白IAPs与caspase-9和caspase3的结合，实验中发现ANTP-SmacN7可以有效地提高细胞的辐射敏感性。

我们的研究显示，ANTP-SmacN7作为一个IAPs的蛋白抑制剂，在H460细胞系中具有明显的辐射增敏效应，这表明ANTP-SmacN7可以作为一种有效的辐射增敏剂。本研究的焦点是通过转导外源性的Smac蛋白模拟物进入细胞，从而发挥促凋亡作用，为了既能保证有Smac蛋白的活性，又能保证可以穿透细胞^[13, 14]^，我们在SmacN7序列基础上又连了1个ANTP穿膜序列，形成其融合肽。我们的实验结果也证实ANTP-SmacN7和细胞共培养后即可穿透细胞膜并进入细胞。虽然ANTP-SmacN7单独作用对H460细胞的辐射增敏效果不明显，但是在联合放射的基础上，通过细胞克隆实验和流式细胞术实验证明ANTP-SmacN7可以起到明显地促进辐射诱导的细胞凋亡的作用。以往的研究也表明，IAPs在正常情况下，与caspase3结合而使其丧失活性，从而抑制细胞凋亡^[15]^，Smac在正常细胞不诱发凋亡，而给予凋亡刺激（包括抗肿瘤药物、电离辐射和化学信号等）才能使其特异地作用于IAPs，解除IAPs与caspase3的结合，激活caspase3，促进细胞凋亡^[3]^，而且其促凋亡作用也与细胞受到凋亡信号刺激后，细胞色素C的释放有关^[16]^。本研究也发现，ANTP-smacN7虽然对caspase3蛋白表达水平不产生影响，但可增加cleaved caspase3的量，表明ANTP-smacN7促进了caspase3蛋白的活化，ANTP-smacN7与IAPs结合后，抑制了IAPs与caspase3的结合，阻断了IAPs的抑制凋亡活性，间接地促进了caspase3的活化，进而促进细胞凋亡。因此，得出的结论是ANTP-SmacN7对H460细胞具有辐射增敏作用，辐射增敏比达到了1.86，尤其对IAPs高表达的辐射抗的细胞更为合适。

另外，ANTP-SmacN7融合肽在促进细胞凋亡过程中对凋亡信号级联的影响尚不明确。有研究^[17-19]^表明，caspase活化在Smac提高辐射诱导的细胞凋亡中起着关键的作用。ANTP-SmacN7融合肽对caspase-8和caspase-9的表达及其活性有何影响？这有待我们在下一步的研究中深入探索。总之，本研究证实了ANTP-SmacN7是一种有效的辐射增敏剂，为ANTP-SmacN7作为一种新型的肿瘤放疗增敏药物提供了科学的理论基础。
